# Prioritizing conservation actions in urbanizing landscapes

**DOI:** 10.1038/s41598-020-79258-2

**Published:** 2021-01-12

**Authors:** A. K. Ettinger, E. R. Buhle, B. E. Feist, E. Howe, J. A. Spromberg, N. L. Scholz, P. S. Levin

**Affiliations:** 1 The Nature Conservancy- Washington, Seattle, WA 98121 USA; 2 Northwest Fisheries Science Center, National Marine Fisheries Service, National Oceanic and Atmospheric Administration, 2725 Montlake Blvd E, Seattle, WA 98112 USA; 3 Biomark Applied Biological Services, 705 S 8th St, Boise, ID 83702 USA; 4 University of Washington, Seattle, WA USA

**Keywords:** Ecology, Conservation biology, Restoration ecology, Urban ecology

## Abstract

Urbanization-driven landscape changes are harmful to many species. Negative effects can be mitigated through habitat preservation and restoration, but it is often difficult to prioritize these conservation actions. This is due, in part, to the scarcity of species response data, which limit the predictive accuracy of modeling to estimate critical thresholds for biological decline and recovery. To address these challenges, we quantify effort required for restoration, in combination with a clear conservation objective and associated metric (e.g., habitat for focal organisms). We develop and apply this framework to coho salmon (*Oncorhynchus kisutch*), a highly migratory and culturally iconic species in western North America that is particularly sensitive to urbanization. We examine how uncertainty in biological parameters may alter locations prioritized for conservation action and compare this to the effect of shifting to a different conservation metric (e.g., a different focal salmon species). Our approach prioritized suburban areas (those with intermediate urbanization effects) for preservation and restoration action to benefit coho. We found that prioritization was most sensitive to the selected metric, rather than the level of uncertainty or critical threshold values. Our analyses highlight the importance of identifying metrics that are well-aligned with intended outcomes.

## Introduction

Limited resources necessitate prioritizing where, when, and how conservation actions occur. Indeed, prioritization, optimization, and, to some extent, triage are at the core of conservation planning, and numerous approaches exist for prioritizing conservation actions^1–3^. These include ecosystem-based risk assessment^4,5^, capitalizing on ecosystem services^6^, cost-effectiveness approaches that seek to maximize the conservation return on investment^7–9^, and hybrid methods, such as those that incorporate both cost-effectiveness for a target benefit and ecosystem services as co-benefits^10^.

Although conservation prioritization is widely studied and implemented, few examples exist in urban settings. Yet, effectively and efficiently allocating conservation resources in urban and urbanizing areas is imperative. Urban areas are growing around the world: most humans now live in cities and urban populations continue to rise rapidly^11,12^. With expanding urban areas, the need for conservation is heightened because habitat area, quality, and connectivity decline as habitat fragmentation and species use of urban areas increase^11,13^. Moreover, the co-benefits of urban biodiversity conservation are widely acknowledged, ranging from reducing urban heat islands and air pollution to improving human health^14–17^. Prioritizing conservation action is particularly important in and near cities, where real estate values are high and there are often many competing interests for urban lands.

One transformative change in land-use associated with urbanization is expanding imperviousness from pavement, roofs, and other hard surfaces^18,19^. Among other things, this change alters the quality, quantity, and routing of surface water runoff as it moves across the landscape during and after rain events. Urban stormwater flows into stream and river networks via drainage pipes and open ditches, reducing habitat quality for diverse organisms^20^. Urban waterways around the world exhibit a “syndrome” characterized by high flow velocities, high concentrations of nutrients and synthetic contaminants, and reduced species richness compared to less developed areas^21–24^.

Future conservation prospects for urban watersheds may be bolstered by geographically broad, intensive efforts to reduce urban stormwater’s multiple impacts. Conservation actions to mitigate stormwater impacts range from conventional land preservation, common in more rural areas^25^, to innovative green stormwater methods to slow and infiltrate surface runoff through various combinations of plants, soils, and other materials^26^. Regardless of the approach, the overarching goals are to reduce both pollution (by capturing toxics) and flooding in receiving waters. Soil infiltration is an effective means of removing contaminants and preventing toxicity in coho salmon and other aquatic species^27–29^. Implementing green stormwater infrastructure is costly and time-consuming, especially in the built environment, however. This and other restoration techniques, such as constructed wetlands^30^*,* cannot reasonably be implemented everywhere and therefore must be prioritized in some way.

Another challenge for conservation decision-making in urban (and other) settings is the need for up-to-date, detailed ecological data, which can affect prioritization and planning outcomes^31,32^. Available data and quantitative analyses are rarely rigorous enough to identify specific numerical targets for all priority populations and communities^33^. There may also be limited returns on the collection of more data, given inevitable constraints on time, personnel, and financial resources. Due to the urgency of many environmental threats, it is frequently necessary to implement conservation actions with incomplete biological data^34^. It is unclear the extent to which uncertainty in biological information might affect prioritization of conservation action in urbanizing landscapes.

**Figure 1:**
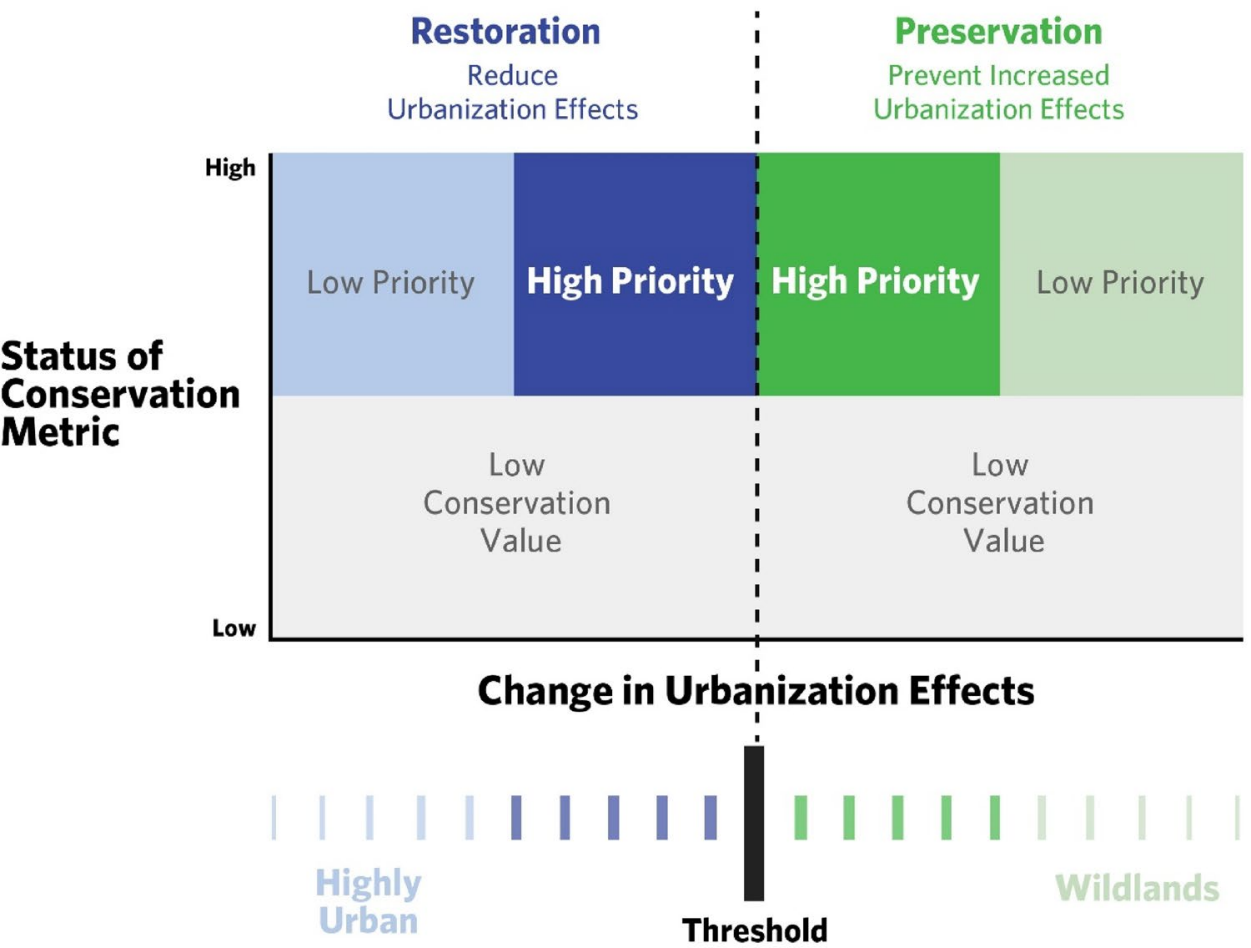
Conceptual framework for prioritizing restoration and preservation actions, using an ecological or utility threshold (dashed black line) to distinguish between restoration locations (blue, where negative effects of urbanization need to be reduced to reach the threshold) and preservation locations (green, where negative effects of urbanization should be prevented to avoid reaching the threshold). The prioritization is guided by the location of the threshold and the status of a relevant conservation metric. Locations with a high status of the conservation metric and those closer to the threshold are higher priority for conservation action (darker shades) compared to those further away (lighter shades) because the *x*-axis is a proxy for effort. Restoration sites close to the threshold require less effort to mitigate the effects of urbanization to an appropriate level compared to more urban sites (far left). Preservation sites close to the threshold are at greater risk of exceeding the threshold than less developed sites and are therefore higher priority for preservation action. In our case study, the ecological threshold is the level of urbanization effects associated with the critical level of coho pre-spawn mortality (*M*_crit_), and we evaluate two different conservation metrics: amount of habitat for coho and for Chinook. A high conservation metric status equates to a large amount of habitat, whereas low status equates to little habitat.

Here, we modify and extend a cost effectiveness framework for prioritizing conservation actions (e.g.,^35–37^) in data-limited urban and urbanizing settings. Our goal is to develop a general framework for conservation in urban and urbanizing areas (Fig. 1) that is grounded in the best available data, and incorporates uncertainties associated with incomplete ecological data and rapidly changing landscapes. Our framework requires three components: (1) the status of a conservation metric (e.g., habitat quantity); (2) the cost or effort associated with mitigating threats to that metric; and (3) an ecological or utility threshold. An ecological threshold is a point at which small changes in environmental conditions produce large and sometimes abrupt, responses in a conservation target, whereas a utility threshold refers a point at which small changes in environmental conditions produce substantial changes in the societally desired state of a system^38,39^. Using these three components, we seek to minimize cost or effort while reducing the risk of crossing such thresholds, and thus causing substantial declines in the state of conservation metrics (c.f. ^36^).

In urbanizing landscapes, achieving conservation goals typically requires either restoration of the built environment (e.g., green storm infrastructure, constructed wetlands) or preservation of undeveloped wildlands (e.g., preventing development through establishing preserves or conservation easements). Our framework explicitly considers both restoration and preservation as potential conservation strategies, and we create separate prioritizations under each (Fig. 1, see “Methods” for details), as these different strategies constitute distinct actions. We operationalize this framework and explore three questions:

1. How does altering the conservation objective, and thus the focal metric, change the locations that are prioritized for conservation action (i.e., preservation or restoration)?
2. How do projected landscape changes (future population growth, development) modify these priorities?
3. How does altering selected thresholds and/or acceptable uncertainty change the locations that are prioritized for conservation action?

**Figure 2:**
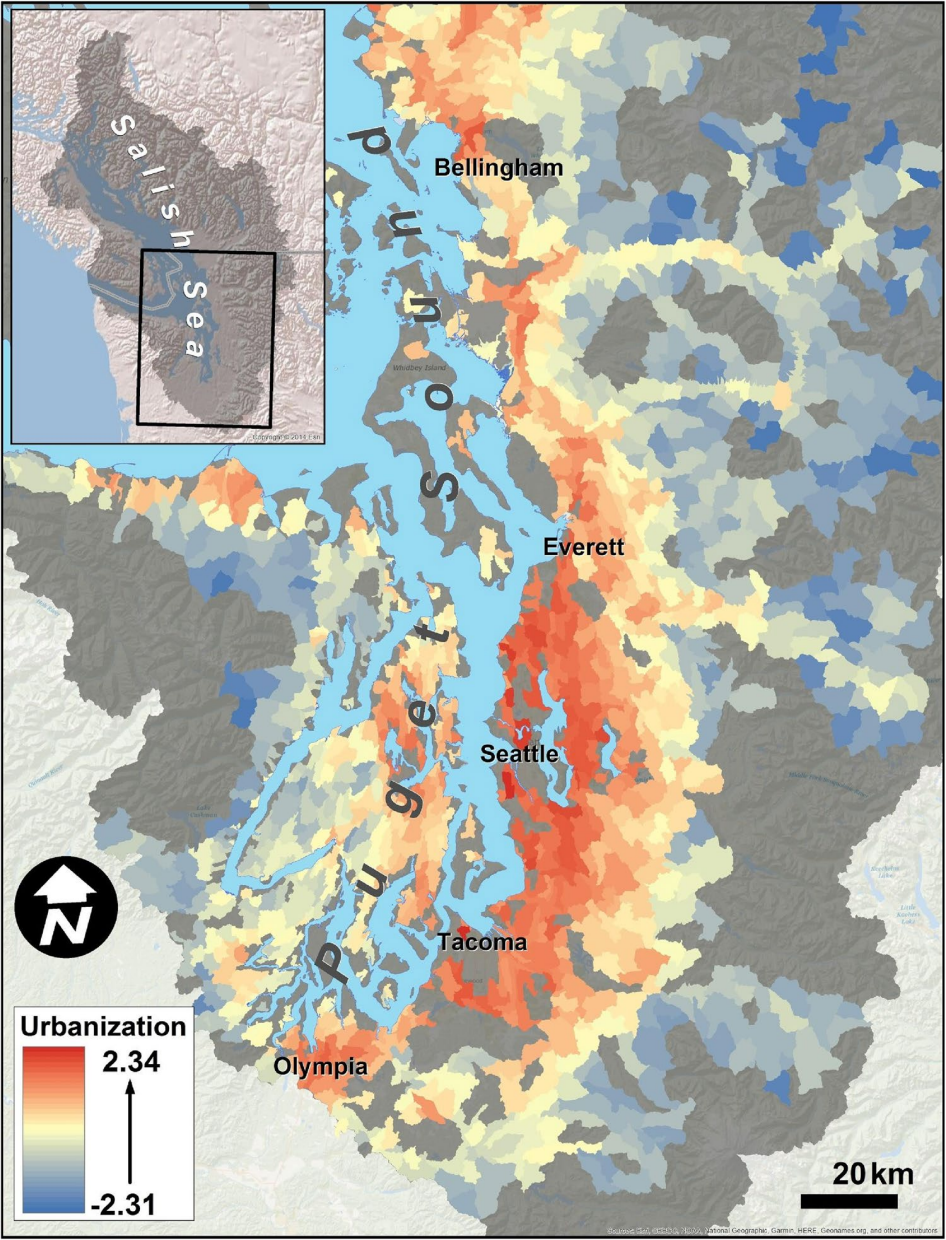
Gradients of urbanization across watersheds in the Puget Sound Basin. Urbanization represents the combined effects of 19 land cover (e.g., higher imperviousness) and land use (e.g., increasing motor vehicle traffic) attributes on coho pre-spawn mortality risk, estimated using a Bayesian structural equation model^42^. The degree of urbanization ranges from relatively remote areas (blue) to highly developed (red). Map generated using Esri ArcMap software (v. 10.1, https://www.esri.com/en-us/home).

As a case study, we apply our framework to identify priority areas of preservation and restoration in Puget Sound, Washington State, USA (Fig. 2), which spans upland, freshwater, estuarine and marine habitats encompassing Seattle and extending from Olympia, Washington to the Canadian Border. The region is home to over 4.2 million people, with projected human population increases of 2 million by 2050^40^. This rapid growth, combined with the more than 40 species listed as threatened, endangered, or candidates for state and federal protection, make Puget Sound an important focal area for prioritizing conservation action^41^.

**Figure 3:**
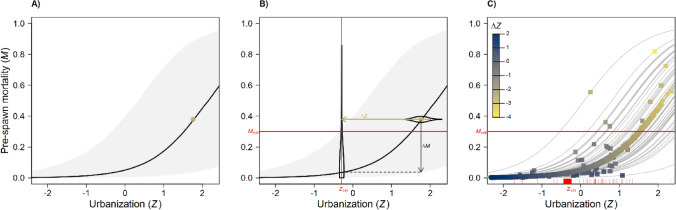
Predicted pre-spawn mortality (*M*) increases with urbanization, represented by the latent factor *Z* in the hierarchical Bayesian structural equation model of ^42^. (**A**) shows the posterior median logistic regression function (black curve) and associated 90% credible interval for a site whose current conditions (*Z*_cur_ and *P*[*M* | data]) are shown as a point estimate. (**B**) Given this relationship and a specified critical mortality risk (*M*_crit_ = 0.3, horizontal red line), we estimated the level of urbanization (*Z*_crit_, vertical red line) that gives a probability 1 – *α* of exceeding *M*_crit_ (here *α *= 0.9). The change in urbanization required to bring *Z*_cur_ (uncertainty indicated by the horizontal violin plot) below *Z*_crit_ with probability *α* is Δ*Z* (horizontal arrow), and the reduction in median *M* risk associated with this change is Δ*M* (vertical arrow). (**C**) Δ*Z* is thus a proxy for restoration effort (Δ*Z* < 0) or preservation resilience (Δ*Z* > 0). Points indicate posterior median estimates of current conditions in subbasins with observed mortality data used to fit the model (squares) and out-of-sample subbasins used for mapping and prioritization (circles). Gray lines show the subbasin-specific regression functions.

We address two conservation objectives (1) habitat for coho salmon, which are highly sensitive to toxic urban runoff and frequently exhibit high mortality among adults returning to spawn in urban streams (i.e., high pre-spawn mortality^42,43^), and (2) habitat for Chinook salmon (*O. tshawytscha*), which are protected under the U.S. Endangered Species Act. The effect of urbanization (represented by *Z*, a latent factor summarizing multiple landscape attributes such as percent cover imperviousness, traffic, and forest cover at the watershed, or subbasin^44^, level) on coho salmon pre-spawn mortality has been quantified previously by hierarchical Bayesian structural equation modelling^42^ (Fig. 3). Separate research has identified that current rates of coho spawner mortality in Puget Sound urban streams are likely to drive otherwise healthy coho populations to local extinction on a timescale of a few decades^45^. We term this “critical pre-spawn mortality threshold” (*M*_crit_) and use a 30% mortality value to identify the ecological threshold in our case study: critical urbanization value, *Z*_crit_, the level of urbanization corresponding to a given *M*_crit_ (Fig. 3). *Z*_crit_ varies by subbasin and is not associated with fixed conditions across all subbasins (e.g., 10% imperviousness); we use a published model^42^ to estimate *Z*_crit_ for each subbasin in the Puget Sound region. We quantify for each subbasin the difference between *Z*_crit_ and the current level of urbanization effects (*Z*_cur_); we term this difference ΔZ, or the desired change in urbanization effects. Using our framework (Fig. 1), we examine the sensitivity of prioritization to uncertainty in ΔZ and *M*_crit_, as well as to two different conservation metrics (amount of habitat for coho versus amount of habitat for Chinook salmon; see “Methods” for details).

## Results

We applied our cost-effectiveness framework, which uses required change in urbanization effects (Δ*Z*) as a proxy for effort, to examine how altering the conservation objective (from coho to Chinook), as well as shifting thresholds of *M*_crit_ and acceptable uncertainty, modify the locations that are prioritized for conservation action.

### Change in prioritization outcome based on the conservation metric

**Figure 4:**
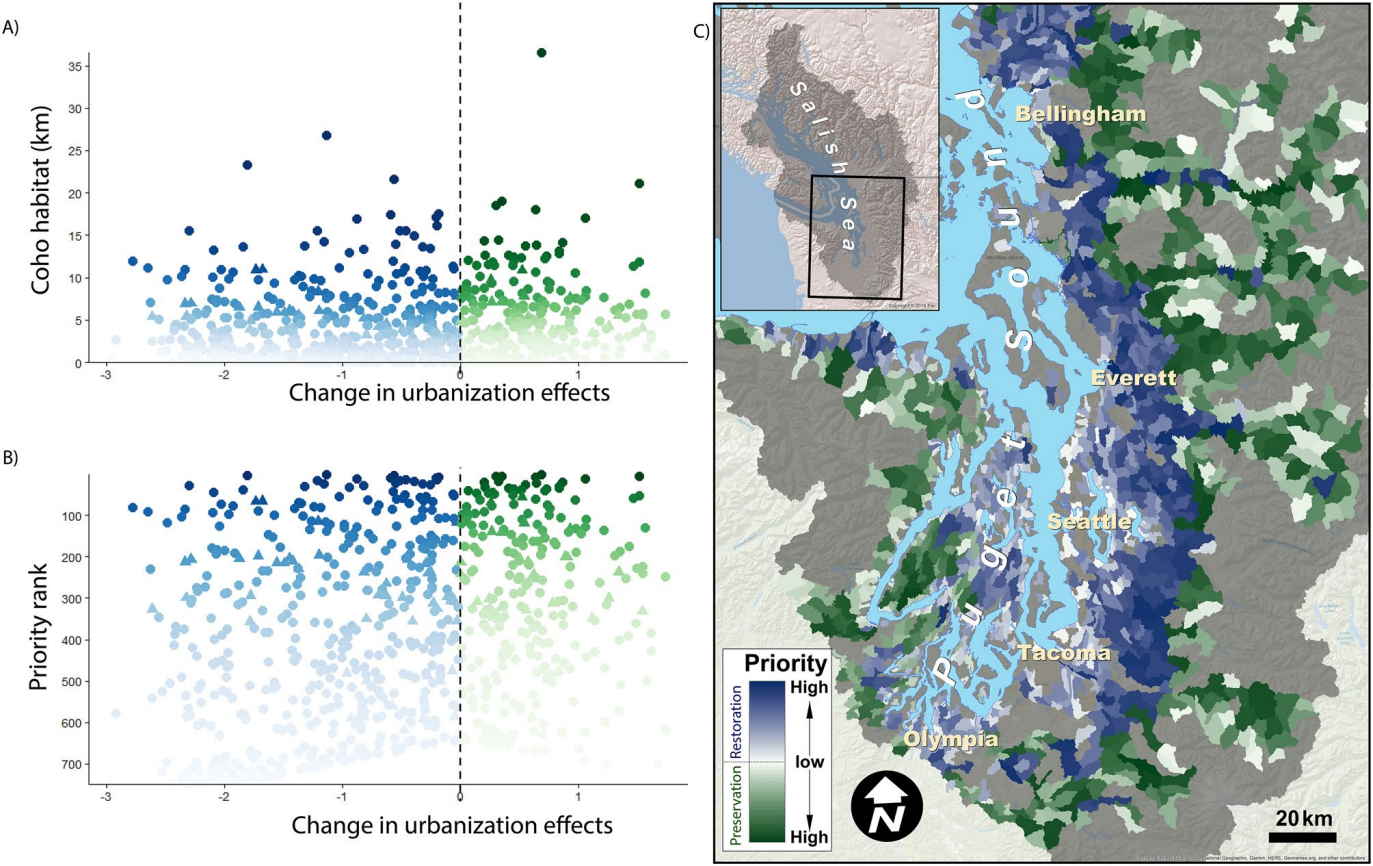
Prioritization of subbasins, using amount of coho habitat as the conservation metric. Subbasins in need of restoration (i.e., Δ*Z* < 0, *N* = 953) are shown in blue; those that are candidates for preservation (Δ*Z* > 0; *N* = 449) are shown in green, with darker colors representing higher priority. We calculate prioritization scores for each subbasin shown in (**A**) and use scores to rank all locations. For both restoration and preservation subbasins, a lower prioritization score is associated with higher priority (see “Prioritizing sites for conservation or restoration action” in “Methods”). Subbasins are ranked by priority on the *y*-axis in (**B**) (1 = top priority). (**C**) shows subbasin locations. Coho habitat is quantified as kilometers of stream within the subbasin. Map generated using Esri ArcMap software (v. 10.1, https://www.esri.com/en-us/home).

**Figure 5:**
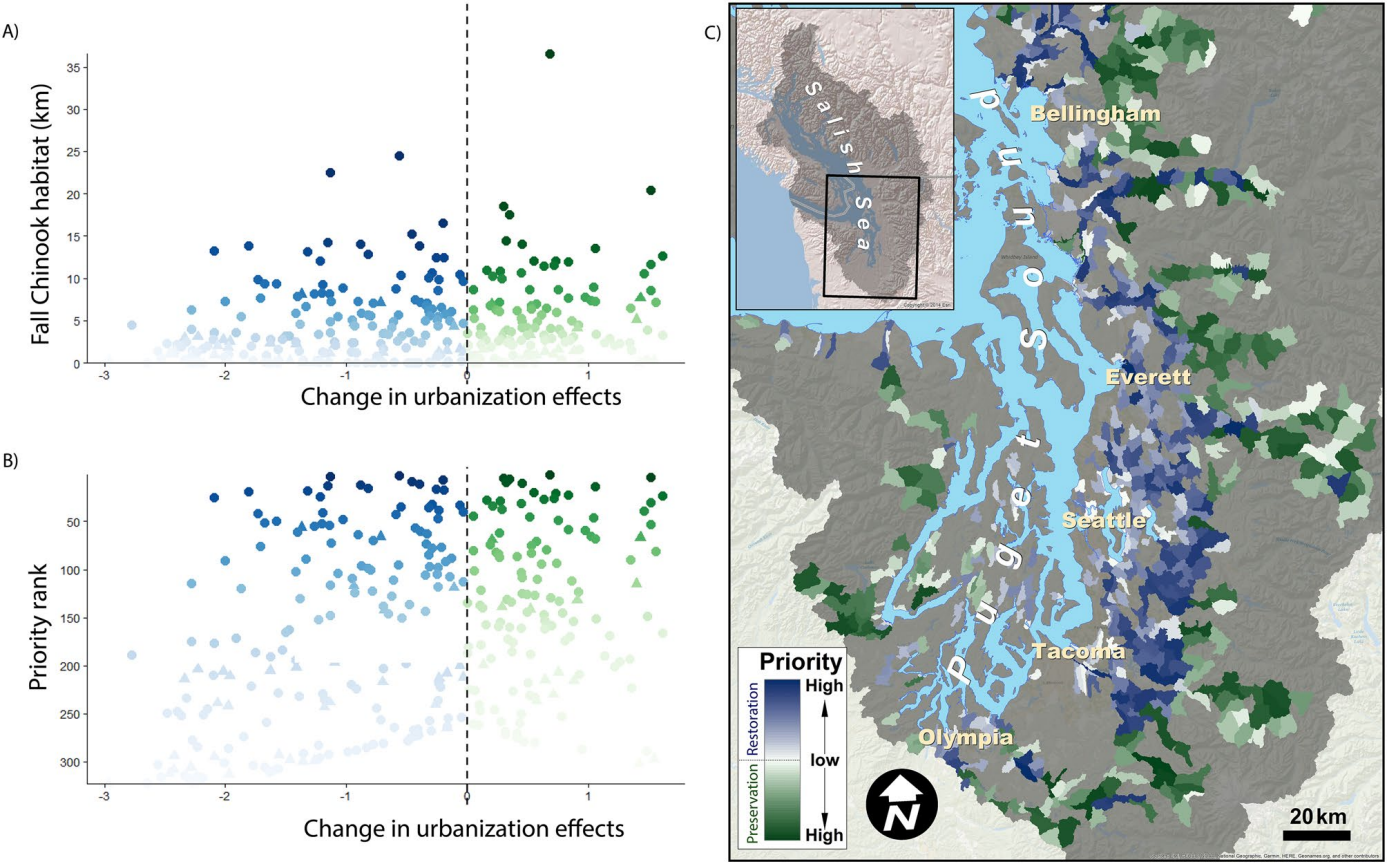
Prioritization of subbasins, using amount of fall Chinook habitat as the conservation metric. Subbasins in need of restoration (i.e., Δ*Z* < 0, *N* = 479) are shown in blue; those that are candidates for preservation (Δ*Z* > 0; *N* = 195) are shown in green, with darker colors representing higher priority. We calculate prioritization scores for each subbasin shown in (**A**) and use scores to rank all locations. For both restoration and preservation basins, a lower prioritization score is associated with higher priority (see “Prioritizing sites for conservation or restoration action” in “Methods”). Subbasins are ranked by priority on the *y*-axis in (**B**) (1 = top priority). (**C**) shows subbasin locations. Chinook habitat is quantified as kilometers of stream within the subbasin. Map generated using Esri ArcMap software (v. 10.1, https://www.esri.com/en-us/home).

Across preservation and restoration sites (Figs. 4, 5), our framework generally prioritized subbasins with intermediate urbanization effects (i.e., sites with Δ*Z* values closer to zero, which are neither very urban nor very rural). High priority subbasins varied, depending on the conservation metric. For example, coho habitat was more widespread than fall Chinook habitat in the Puget Sound region and many locations prioritized for coho do not provide habitat for Chinook (Figs. 4B, 5B, Table S1, S2). Some subbasins were consistently identified as high priority across both metrics, however. For example, subbasins 296 and 15 were ranked priority 1 and 2, respectively, using both conservation metrics (Table S1, S2).

### Implications of future urbanization

**Figure 6:**
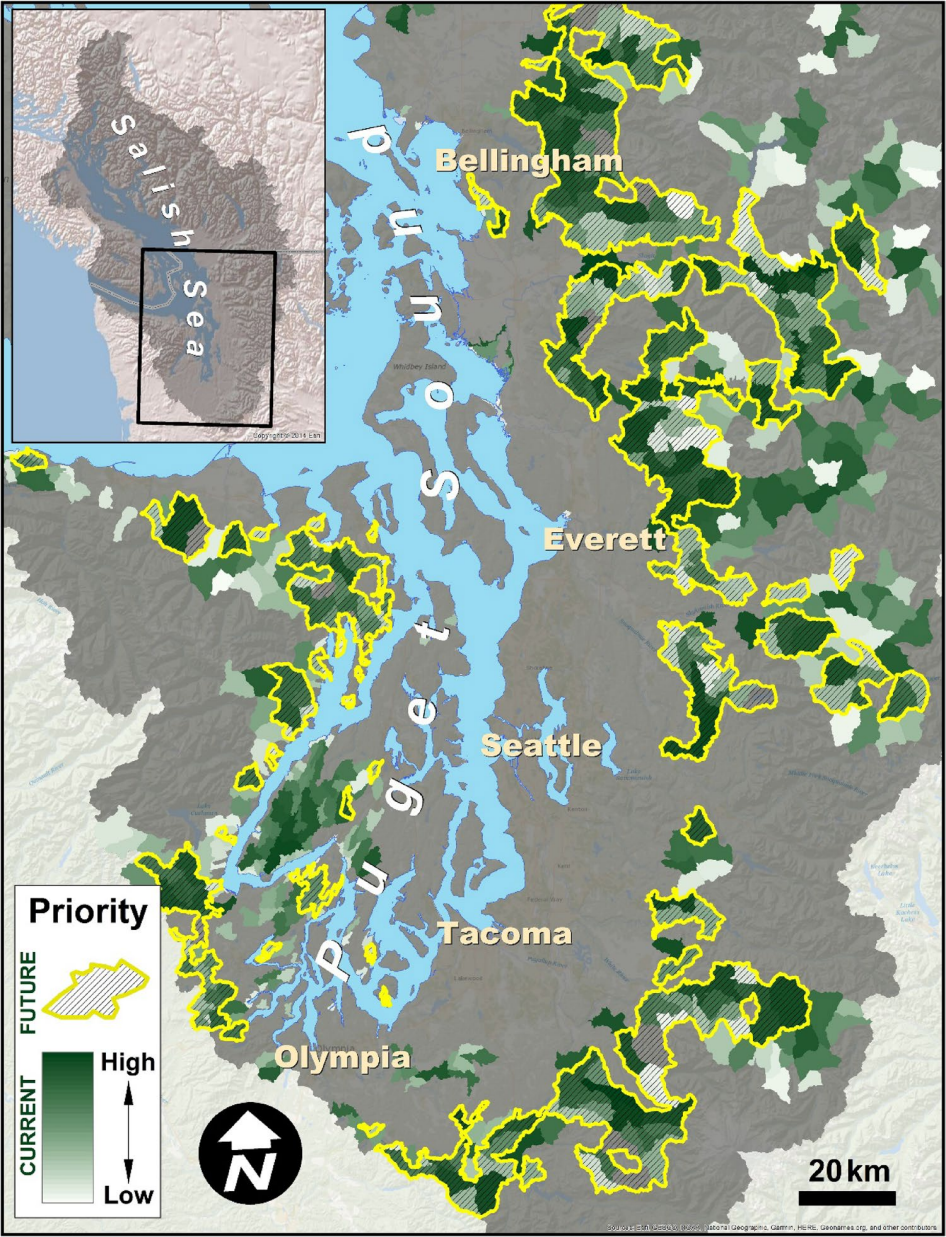
Expected development will increase urbanization impacts to coho habitat. Many subbasins currently identified as high-priority for preservation action (i.e., because predicted pre-spawn mortality is low, shaded in green with darker colors signifying higher priority, as in Figs. 4, 5) are projected to experience ≥ 10% imperviousness by the year 2060 under a status quo future scenario^46^, likely increasing pre-spawn mortality above the critical value of 0.3 (areas outlined in yellow with shading). Map generated using Esri ArcMap software (v. 10.1, https://www.esri.com/en-us/home).

Forecasted rates of development in the region suggest that many subbasins not currently experiencing high rates of coho spawner die-off will undergo substantial urbanization in the coming decades, which may push coho spawner mortality rates above critical levels by 2060. At present, there are about 11,000 km^2^ of subbasins identified as benefitting from preservation (i.e., Δ*Z* > 0) and with current mean imperviousness < 10%. By the year 2060, predicted future development is likely to increase imperviousness above 10% for many subbasins (Fig. 6), depending on the future development scenario that plays out. Under the “status quo” scenario, ~ 32% of that 11,000 km^2^ requiring preservation is predicted to experience > 10% imperviousness, placing those subbasins at risk (Fig. 6). For the less aggressive “managed growth” scenario and the more aggressive “unconstrained growth” scenario, the proportions of those preservation areas that will be at risk are ~ 16% and ~ 42%, respectively.

### Effects of altering thresholds and uncertainty

**Figure 7:**
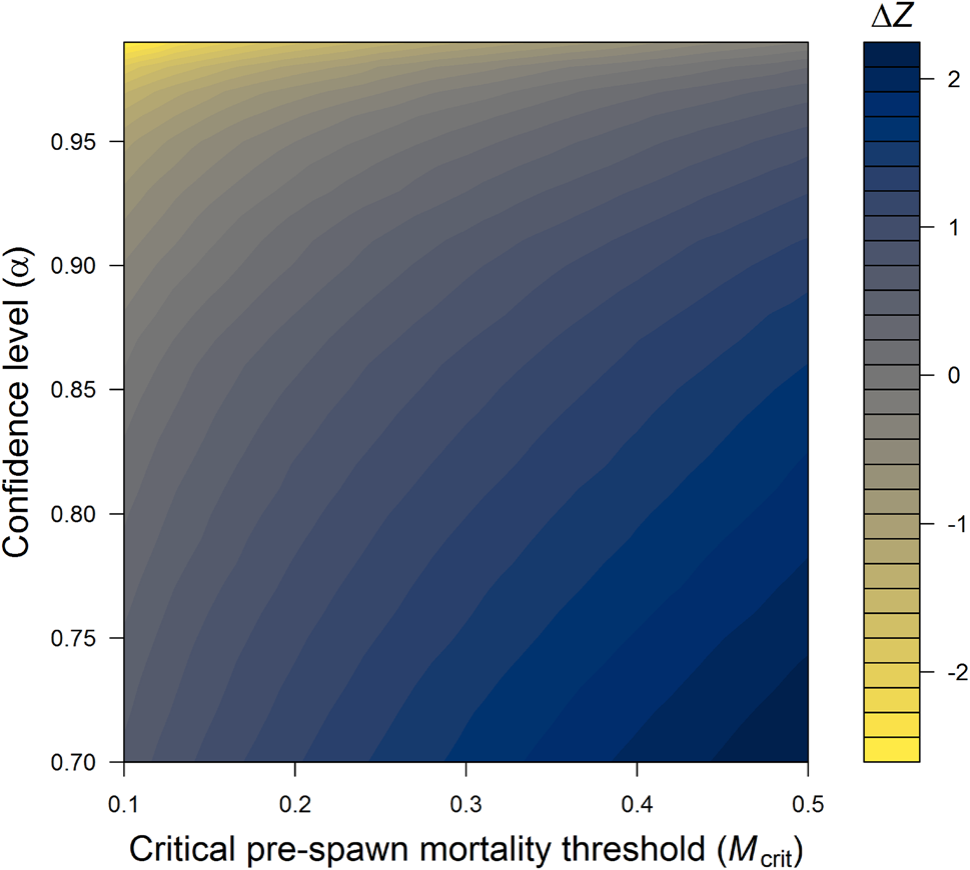
Estimates of restoration effort or preservation resilience (Δ*Z*) vary with uncertainty, i.e., the critical level of pre-spawn mortality (*M*_crit_) and the desired uncertainty level (*α*), as shown here for one example subbasin (#845). The change in urbanization relative to current conditions (Δ*Z*) needed to ensure a predicted mortality risk no greater than *M*_crit_ with probability *α* is shown for an arbitrary site. In this example, the site may fall into either the restoration (Δ*Z* < 0) or preservation (Δ*Z* > 0) category depending on the criteria specified. More stringent criteria (lower *M*_crit_ or higher *α*) result in lower resilience or greater restoration effort (i.e., lower Δ*Z*).

**Figure 8:**
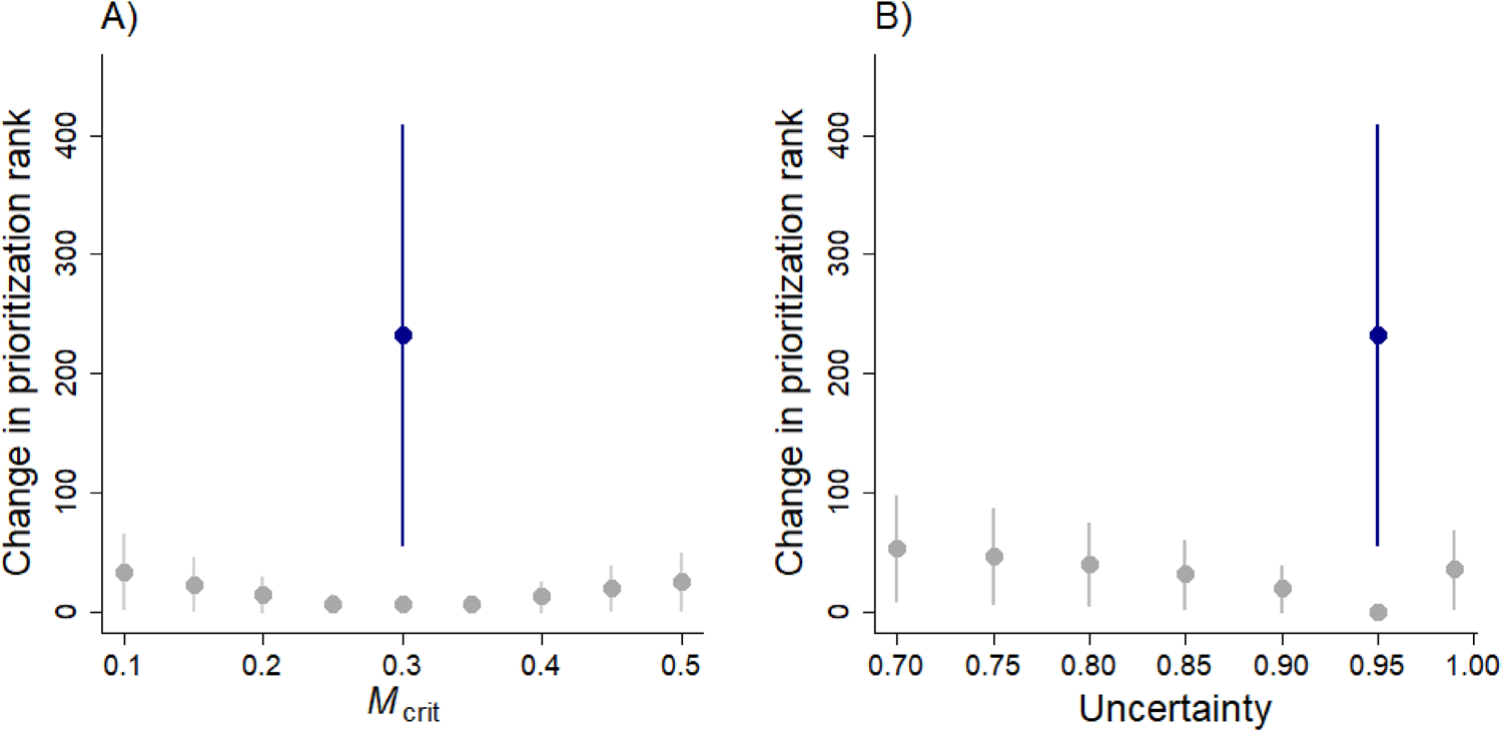
Shifting to a different conservation metric alters prioritization (represented in blue) more so than assuming a different *M*_crit_ (in gray, **A**) or acceptable uncertainty level (*α*) (in gray, **B**). We show the mean change in priority rank, compared to using a metric of coho habitat, with *M*_crit_ = 0.3 and *α* = 0.95. Blue dots show the mean change in priority rank when shifting from a metric of coho habitat to a metric of Chinook habitat (with *M*_crit_ = 0.3 and *α* = 0.95).

Altering selected thresholds for *M*_crit_ and acceptable uncertainty levels (*α*) associated with current urbanization levels (*Z*) led to shifts in estimates of Δ*Z* (see Fig. 7 for one example subbasin). In addition, varying *M*_crit_ and *α* altered prioritization, but the effect was minor in comparison to changing the focal conservation metric from coho habitat to Chinook habitat. Varying the level of *M*_crit_ (e.g., from 0.2 to 0.4) and altering *α* affected the number of subbasins identified as needing restoration versus preservation action (e.g., Fig. 7), and altered the prioritization ranking (Fig. 8). More stringent criteria (lower *M*_crit_ or higher *α*) result in lower Δ*Z* (i.e., reducing resilience for preservation sites and increasing restoration effort for restoration sites). Thus, a lower *M*_crit_ or higher acceptable level of uncertainty (*α*) would raise the priority of a preservation site (moving it closer to the threshold, Fig. 1), and would lower the priority of a restoration site (moving it further from the threshold).

## Discussion

Conservation practitioners often confront conflicting demands. On one hand, conservation accomplishments may be evaluated by hectares preserved, kilometers of stream restored, or gallons of water cleaned^47–49^, potentially incentivizing the acquisition or restoration of large areas that are inexpensive or easy to restore. In contrast, some have argued that conservation must focus on regions with the highest conservation benefit, a strategy illustrated by the “Global Deal for Nature”^50^. Given the finite public and private funds that realistically can be directed towards conservation, however, a practical solution to the conflict facing practitioners is a focus on conservation efficiency measured as the conservation cost effectiveness or return on financial investment^9,35^.

We used a cost-effectiveness framework for prioritizing conservation action in urbanizing areas and applied it to an urgent problem, coho salmon urban pre-spawning mortality syndrome in the Puget Sound region of Washington State, USA. This study builds on a large and important foundation of work quantifying conservation priorities (such as^1,33^) in at least two major ways. First, it encompasses both restoration and preservation solutions; previous work usually focuses on preservation only (e.g., identifying locations for new preserves^51^). Second, we adapted the general cost-effectiveness approach for conservation in urban and urbanizing areas, which have largely been ignored by other conservation prioritization schemes. Given the rapid growth of urban areas, the potential threats they pose for biodiversity conservation, and the relative difficulty of conducting conservation in urbanizing areas, the expansion of conservation prioritization frameworks to cities and surrounding areas is crucial^11,12^.

Our analysis highlights why threat maps alone are insufficient to guide conservation action^52^: incorporating focal conservation targets or metrics is critical and conservation priorities will vary, depending on the selected metric^53^. Maps of threats such as urbanization effects (Fig. 2) or coho pre-spawn mortality^42^ are important starting points but cannot be translated into conservation action in a straightforward manner. For example, should a subbasin with extremely high predicted pre-spawn mortality be prioritized for restoration? Perhaps not, if the amount of habitat present is very small. Our approach combines the threat with a clear, quantifiable objective^52^, such as amount of coho habitat (Fig. 4) or fall Chinook habitat (Fig. 5), to prioritize action, using a framework similar to a return-on-investment approach^54^. We find that the identity of the selected conservation metric dramatically alters which sites are prioritized (compare, e.g., Fig. 4 to Fig. 5; see also Table S1, S2), and has a far greater effect on prioritization than altering acceptable levels of uncertainty or *M*_crit_ (Fig. 8). We chose two different metrics as examples: coho habitat and Chinook habitat (Figs. 4, 5). Selecting an entirely different metric, such as number of people with access to healthy stream habitat (if the conservation goal was related to human health and well-being) would likely yield a completely different prioritization. This demonstrates the value of quantitative prioritization tools that incorporate biological attributes and uncertainty, even with limited biological data, because trade-offs of different targets become apparent^54,55^.

Our focus in this case study was prioritizing conservation actions to mitigate the toxicity of urban runoff, as coho salmon are extremely sensitive to this threat^56^. The lethal response of coho to untreated urban runoff is so acute that, in the Pacific Northwest, this species is an established sentinel species for both field-based monitoring of degraded water quality^43^ and green infrastructure effectiveness^27,29^. We note, however, that problems facing urbanizing ecosystems as a whole go beyond water quality. Urban stream syndrome encompasses physical characteristics such as altered stream morphology and hydrographs, as well^20,23,24^. Salmonid species face numerous additional threats, including novel nonnative species, hatchery practices, and climate change^34,55,57,58^. The framework we developed here could be modified to accommodate these and other threats, by adding the necessary geospatial data layers, making it applicable to a range of conservation challenges in urbanized settings.

One such challenge is habitat connectivity, long-considered a cornerstone of watershed restoration^59,60^. Connectivity is typically framed in the context of hydrologic processes and fish passage (e.g., adequate flow, migration barriers); there are also complex relationships between human activities, upland changes in land use, rainfall patterns, surface runoff, and degraded water quality along a gradient of stormwater inputs to river and stream networks^61^. Here we have summarized effects of directional connectivity on water quality at a watershed resolution (i.e., landscape covariates accounted for upstream conditions within the subbasin), as investments to reduce non-point source pollution at upstream sites within a watershed are likely to benefit downstream aquatic communities. We focus on connectivity within (not between) subbasins in our framework because the acutely toxic response of coho salmon to landscape conditions and stormwater contaminants within the watershed are well-documented^28,42,62^. Nevertheless, migratory species, such as salmonids, do use a mosaic of interconnected watersheds at different points in their life history. Thus, there may be less benefit to some species if restoration and preservation strategies produce a scattershot suite of locally “cost efficient” restoration sites than if the broader landscape of functionally connected habitat corridors are incorporated into planning^63^. For coho, this might involve, for example, incorporating knowledge of source and sink populations^45^. Our prioritization framework and analysis could be modified to include connectivity across watersheds, using standard approaches to penalize sites based on fragmentation^64,65^ and promote sites known to be ecologically significant to target species^63^.

Our analysis is based on a quantitative relationship between urbanization and pre-spawn mortality of coho salmon, with mortality increasing in concert with urbanization in Puget Sound (Fig. 3). Dominant approaches to stormwater management in the region likely drive this relationship. However, green stormwater infrastructure—designed to spread, infiltrate, and decontaminate stormwater—is intended to mitigate the multifaceted negative effects of urbanization (summarized by Z in our modelling approach) on coho spawner mortality. If green stormwater infrastructure is applied at sufficient intensity in a subbasin, holding other urbanization effects constant, we would expect coho spawner mortality to decrease in that subbasin (though there is uncertainty in biological responses following restoration). That is, green stormwater infrastructure is designed to break correlations in the existing data used to parameterize our structural equation model^42^. Observed declines over time in coho pre-spawn mortality at particular subbasins with extensive green infrastructure, or a shift in the correlation structure between coho pre-spawn mortality rates and landscape indicators of urbanization, may be effective stormwater monitoring diagnostics that would facilitate identification of how much green infrastructure is sufficient to benefit coho.

Our work here is not an endpoint, but an example of a further step in the management decision-making process. It may be seen as part of a structured decision-making process for choosing among alternative management actions^66,67^. Restoration actions that could be considered include green stormwater infrastructure techniques^68^, low-impact development, such as compact development with reduced imperviousness^69^, street sweeping^70^, and constructed wetlands^30^. Our framework could be used by managers to choose among alternative locations in which to invest in stormwater intervention projects. Of course, there are many complexities to implementing our framework on the ground, beyond uncertainty in the relationship between urbanization and coho mortality (Fig. 3). A subbasin identified as high priority for conservation would require landowner contact, site visits, additional ecological assessment and permitting, and, ideally, a monitoring and adaptive management plan, prior to implementing any action. Our experience is that urban natural resource managers often behave opportunistically when implementing projects. This stems partially from the need to coordinate projects and funding across governmental agencies and grant opportunities. As a result, project investments are not always located in the most biologically relevant areas. A structured framework such as the one we present could help managers quickly determine which opportunities are worth pursuing by combining cost-effectiveness with ecological significance.

Our framework prioritizes action where conditions are close to an urbanization threshold, based on the assumption that conservation in these urbanizing areas may achieve the greatest cost-effectiveness. The particular subbasins identified as highest priority for conservation depend on the conservation metric of interest (e.g., coho versus Chinook habitat), but high-priority locations were often in suburban/exurban areas, rather than in either the urban core or in the most rural locations of our focal area. These intermediate locations may be close to an urbanization “tipping point” beyond which coho and other species are unlikely to persist^71,72^. Predicting tipping points has been a focus of conservation and management in a wide range of terrestrial and marine systems^73,74^. A tipping point framework may be useful for conservation in urbanizing landscapes as well, especially if it can be operationalized to identify sites on the cusp of entering an alternative undesirable state^72^. Parts of the globe are urbanizing at a pace even more rapid than our case study of the Puget Sound region (e.g., cities in Africa and Asia^12^); in these areas, our framework may be most useful for identifying high-priority areas for preserving green space from fast-paced urban development. Operationalizing this framework in shrinking cities in the U.S. rust belt^75^, eastern Europe^12^, and elsewhere, on the other hand, may focus more on prioritizing where restorative green infrastructure would yield greater ecological bang for the buck.

To compare cost-effectiveness, we used a proxy for effort (Δ*Z*, the difference between the estimated current urbanization and the subbasin’s critical value for urbanization effects); translating this estimate of effort into financial cost would be complex. Costs are likely to vary widely among the myriad potential conservation actions. For example, retrofitting the built environment (a restoration action) is critical to reducing negative effects of stormwater in urban areas, but is likely more expensive than incorporating clean water strategies into future development plans and limiting growth^55,76^. In addition, costs of preservation vary geographically^72^ and with the approach used (e.g., establishing conservation easements versus preserves^77^). Financial estimates of these different approaches could be incorporated into our framework to prioritize sites for conservation action.

In a rapidly urbanizing world with numerous, intense conservation challenges, the question of where to focus conservation efforts is daunting. Straightforward frameworks, such as the one we use, provide a starting point, but the answer clearly depends on the objectives (and thus the metrics used; Fig. 8), which can be numerous and conflicting. Our approach can be extended to multi-objective prioritization^78^; a fundamental challenge, though, is that funding for conservation actions is uncertain and choosing priority areas and actions requires communities and conservation organizations to define clear conservation goals. Indeed, our work highlights that having the right tools is only beneficial if we can address challenging questions about why we are doing conservation in the first place.

## Methods

### Restoration versus preservation

Our framework creates separate prioritizations for restoration and preservation actions (Fig. 1). We use the term “restoration” to encompass actions that reduce or reverse current negative urbanization impacts on habitats/taxa of concern and the term “preservation” encompasses actions that prevent increases in urbanization impacts. For preservation strategies, our approach considers areas with extremely low levels of development to be a lower priority because these sites are likely to tolerate some increased urbanization without crossing the ecological or utility urbanization threshold. Thus, regions are not prioritized where current urbanization effects are far from the threshold, even though such areas may have high conservation value. In contrast, regions are prioritized for preservation where advancing urbanization results in urbanization effects being near the threshold. Restoration strategies are prioritized in areas where a small change in the urban landscape will reduce negative conditions across a threshold to a preferred state. Restoration strategies that focus solely on ecological outcomes would therefore not be prioritized in highly urbanized locations where conservation metrics are very far from the threshold because the effort associated with restoring conditions up to this threshold is likely prohibitive.

### Metrics

We focus our conservation metrics on Pacific salmon (*Oncorhynchus* spp.), which are culturally, economically, and ecologically important in northwestern North America. They are integral to the culture and traditional practices of indigenous peoples in the region, valued in recreational and commercial fisheries, and are considered keystone species^42,79,80^. We use two Pacific salmon-related metrics for which data are available: amount of coho habitat and amount of Chinook habitat. Within Pacific salmon, we focus particularly on coho, because they have been termed a “sentinel species” for urban stream ecosystems; both juvenile and adult stages are extremely sensitive to water toxicity associated with urbanization^42,43,45,56,81^.

We include a metric for Chinook salmon because many conservation efforts focus on Puget Sound Fall Chinook salmon (e.g., Puget Sound Vital Signs, https://vitalsigns.pugetsoundinfo.wa.gov/VitalSignIndicator/Detail/49), which occur in some of the same watersheds as coho in the region and are listed as Threatened under the Endangered Species Act^82^. Urbanization negatively affects Chinook salmon, but these effects, which include immune suppression and increased susceptibility to disease^83^, are less obvious in this species than in coho. Because relationships between urbanization and Chinook are poorly understood, we could not identify Chinook-specific thresholds for urbanization; we therefore apply the Δ*Z* values developed for coho (see “Estimating site-specific critical pre-spawn mortality and associated urbanization levels” below). In this context, Δ*Z* can be interpreted as change needed to reach a threshold of clean water; it is important to keep in mind that Chinook may have a different tolerance than coho for urban water toxicity and additional research would be necessary to quantify a Chinook-specific threshold. We expect that using a Chinook-specific threshold would further exacerbate differences in locations prioritized across these two metrics.

### Data

For details on land use/land cover and coho pre-spawn mortality data used to model relationships between urbanization and pre-spawn mortality, see^42^. Data layers of coho and fall Chinook use of streams (including documented “rearing”, “spawning” and/or “presence”) were derived from the Washington Integrated Fish Database (WIFD), accessed through Salmonscape (https://apps.wdfw.wa.gov/salmonscape/). These data layers were summarized at the subbasin, or watershed^44^, resolution.

To better understand the future of coho mortality as a function of different urban growth scenarios, we used predicted imperviousness in the year 2060^46^ to quantify the total area of preservation regions that might be at risk under three different development management scenarios: “status quo”, “managed growth” and “unconstrained growth”. These future scenarios differ based on their potential impacts on various indicators of ecosystem function, including population allocation, patterns of urban and rural growth, and protection of sensitive (e.g., wetlands) and nearshore/coastal areas. The three scenarios were generally defined as follows: status quo—no change in approach to constraining urban expansion; managed growth—an aggressive set of management policies that protect and restore ecosystem function and seek to concentrate development within urban growth areas and near regional growth centers; and, unmanaged growth—a relaxation of land use restrictions with limited protection of ecosystem functions.

### Estimating site-specific critical pre-spawn mortality and associated urbanization levels

Our approach relies on estimates of urbanization (Fig. 2) and its predicted relationship with coho pre-spawn mortality (Fig. 3), obtained from a previously developed hierarchical Bayesian structural equation model^42^. That model identified a one-dimensional latent factor (urbanization, *Z*) that summarizes the effects of 19 land use/land cover variables on coho pre-spawn mortality, in conjunction with seasonal precipitation (which we do not incorporate here, setting rainfall equal to its sample mean). We used the model to estimate subbasin-specific critical values (*Z*_crit_) representing the urbanization level at which the posterior probability of exceeding a critical pre-spawn mortality risk (*M*_crit_) equals 1 − *α*, for some specified uncertainty level *α*. The difference between the estimated current urbanization and the critical value (Δ*Z* = *Z*_cur_ − *Z*_crit_, accounting for uncertainty in the posterior distribution of *Z*_cur_), is a proxy for the effort required to restore an impacted site to an acceptable pre-spawn mortality risk (Δ*Z* < 0) or the resilience of a less-impacted (preservation) site to continued encroachment (Δ*Z* > 0).

To find Δ*Z*, we begin with the logistic regression of pre-spawn mortality risk *M* against *Z* from the structural equation model of^42^, which includes a random subbasin-specific intercept (Fig. 3A). We find the critical urbanization level *Z*_crit_ by solving

$$P\left( {M \ge M_{{{\text{crit}}}} | Z_{{{\text{crit}}}} , {\text{data}}} \right) = {1} {-} \alpha ,$$

using the MCMC samples to approximate the posterior cumulative distribution function (Fig. 3B). We then calculate Δ*Z* as the difference between *Z*_crit_ and the *α*-th posterior quantile of the current site-specific urbanization *Z*_cur_, such that

$$P\left( {Z_{{{\text{cur}}}} + \Delta Z \le Z_{{{\text{crit}}}} |{\text{ data}}} \right) \, = \alpha ,$$

as shown in Fig. 3B. We repeated these calculations for a range of reasonable values of *M*_crit_ (0.2–0.4) and *α* (0.8–0.99)*.* Maps presented in the main manuscript use *α* = 0.95 and *M*_crit_ = 0.3, based on^45^, who found that the mean time to extinction for isolated, healthy coho populations experiencing 30% pre-spawn mortality is 70 years.

### Prioritizing sites for preservation or restoration action

We created spatial action maps for conservation action priorities for all subbasins in Puget Sound that support one or more life history stages of coho (sensu^42^). We did this by plotting each focal conservation metric (coho or fall Chinook habitat) against Δ*Z* for each subbasin*.* We calculated the prioritization score as the Euclidean distance between conditions at each subbasin and the maximum value of the focal conservation metric at Δ*Z* = 0, as shown in the equation below and in Fig. S1:

$${\text{score}}_{i} = {\text{dist}}\left( {\left( {{{\Delta }}Z_{i} ,\;{\text{metric}}_{i} } \right),{\text{~}}\left( {0,\;{\text{metric}}_{{{\text{max}}}} } \right)} \right) = \sqrt {{{\Delta }}Z_{i} {\text{~}} + {\text{~}}\left( {{\text{metric}}_{i} - {\text{metric}}_{{{\text{max}}}} } \right)^{2} }$$

Score_*i*_ is the prioritization score for subbasin *i*, Δ*Z*_*i*_ is the difference between the estimated current urbanization at subbasin *i* and *Z*_crit_*;* metric_*i*_ is the value of the focal conservation metric (e.g., km of coho habitat) in subbasin *i;* and metric_max_ is the maximum value of the conservation metric across all subbasins in the analysis. Lower scores indicate higher priority, as they have higher values of the conservation metric (i.e., values closer to the maximum) and Δ*Z* values closer to 0 (corresponding to lower restoration effort or lower resilience to further impacts; Fig. 3). Preservation and restoration were scored and prioritized separately; basins with estimated Δ*Z* < 0 require restoration action, whereas those with Δ*Z* > 0 were prioritized for preservation action.

### Considering future change in imperviousness

We identified subbasins at risk from future development by incorporating predicted coho spawner mortality and percent imperviousness reported by^42^ with predicted imperviousness in the year 2060^46^. Subbasins that had current imperviousness and predicted coho spawner mortality < 10% but were projected to have an imperviousness ≥ 10% in any of the three future scenarios, were tagged as a conservation priority. We chose a 10% threshold for imperviousness based on values in the literature suggesting that streams above this threshold are considered impacted or impaired^84–86^. Subbasins not meeting those criteria were deemed not a conservation priority. Refer to Supplemental Materials for further details.

## Supplementary Information

Supplementary Information 1.

Supplementary Information 2.

## Data Availability

Code for quantifying prioritization scores is available in Appendix 1. Data and code from^42^ that were used to generate coho pre-spawn mortality in Puget Sound may be found online at: http://onlinelibrary.wiley.com/doi/10.1002/eap.1615/full and at https://github.com/ebuhle/cohoPSM. SalmonScape data, used to quantify coho and Chinook habitat are available at https://www.arcgis.com/home/item.html?id=1e56a648718543ab952e75ff9971f086.
