# Supplementary material for: Prioritizing conservation actions in urbanizing landscapes
Source: Sci Rep. 2021 Jan 12;11:818. doi: 10.1038/s41598-020-79258-2 (PMC7804858; doi:10.1038/s41598-020-79258-2)
Supplement: Supplementary file 1 — Supplementary Information 1. [file 41598_2020_79258_MOESM1_ESM.docx]

**Appendix 1 “Prioritizing conservation actions in urbanizing landscapes”** by A.K. Ettinger, E.R. Buhle, B.E. Feist, E. Howe, J.A. Spromberg, N.L. Scholz, P.S. Levin1

**R Code for quantifying prioritization scores**

(stringsAsFactors = FALSE)

library(dplyr)

##d = data frame of spatial data

zplotfx <- function(psm_thresh,attribut){

Zcrit<-min(d$Z_mean[d$p_psm_mean>psm_thresh], na.rm=TRUE)

d$Zcrit<-Zcrit

d$deltaZ<-d$Zcrit-d$Z_mean

attribute<-d[,which(colnames(d)==attribut)]

d$attribut_stan<-(attribute-mean(attribute,na.rm=TRUE))/sd(attribute,na.rm=TRUE)

dxy<-subset(d,select=c(Z_mean,attribut_stan))

score<-as.matrix(dist(rbind(c(Zcrit,max(dxy$attribut_stan,na.rm=TRUE)),dxy), method="euclidean"))[1,-1]

dxy<-cbind(d$ID,dxy,d[,which(colnames(d)==attribut)],score)

dxy<-dxy[-which(is.na(dxy$attribut_stan)),]

dxy<-dxy[order(dxy$score),]

dxy<- data.frame(cbind(dxy,cols))

colnames(dxy)[1:4]<-c("ID","Z","benefit.stan","benefit")

score_cohopres_m<-dxy

}
