# Supplementary material for: Prioritizing conservation actions in urbanizing landscapes
Source: Sci Rep. 2021 Jan 12;11:818. doi: 10.1038/s41598-020-79258-2 (PMC7804858; doi:10.1038/s41598-020-79258-2)
Supplement: Supplementary file 2 — Supplementary Information 2. [file 41598_2020_79258_MOESM2_ESM.docx]

**Supplemental Materials for “Prioritizing conservation actions in urbanizing landscapes”**

A.K. Ettinger*^1,2^, E.R. Buhle^2,3^, B.E. Feist^2^, E. Howe^1^, J.A. Spromberg^4^, N.L. Scholz^4^, P.S. Levin^1,5^

**Supplemental Methods**

Future Scenario and Conservation Prioritization Analysis

In order to better understand potential impacts of future development on coho habitats that are currently less developed in the Puget Sound region, we quantified the total area of habitat currently designated as a preservation priority that would likely be at risk in the future as a function of various development scenarios. We used predicted imperviousness in the year 2060 from Bolte & Vache (2011) under three different development management scenarios: “status quo”, “managed growth” and “unconstrained growth”. These future scenarios differed as a function of the level of management restriction that would be imposed on future development. These restrictions generally focused on where population expansion would be allocated, e.g., within existing growth boundaries, patterns of urban and rural growth, and protection of sensitive (e.g., wetlands) and nearshore/coastal areas. The three scenarios were generally defined as follows (sensu Bolte and Vache 2011): status quo – no change in approach to constraining urban expansion; managed growth - an aggressive set of management policies that protect and restore ecosystem function and seek to concentrate development within Urban Growth Areas (UGA) and near regional growth centers; and, unmanaged growth - a relaxation of land use restrictions with limited protection of ecosystem functions. Initial conditions for the simulations run by Bolte and Vache (2011) were for the year 2001, and they forecasted changes in 10-year intervals out to 2060 using the three aforementioned scenarios. However, we only used the output for the 2060 year in order to capture conditions at the most distant time horizon.

The geospatial data layers (ArcGIS geodatabases) were downloaded from <http://envision.bioe.orst.edu/StudyAreas/PugetSound/> on 2 April 2019. We used the EM_IMPERV attribute for each of the three scenarios, predicted for the year 2060, which was modeled imperviousness as a function of the corresponding development management scenario (Bolte et al 2010).

Process Steps to Calculate Imperviousness in Subbasins (WADOE 2011) Under Various Future Scenarios

1. For each of the three scenarios, status quo, managed growth and unconstrained growth, ran the following (Managed Growth, MG, example):
   1. Converted modeled imperviousness polygons in geodatabase to grid (10m) using ArcCatalog -> ArcToolbox -> Conversion Tools -> To Raster -> Polygon to Raster ->
      1. Input Features = FRAP_ManagedGrowth.gdb -> Year_60_MG
      2. Value Field = EM_IMPERV
      3. Output Raster Dataset = em_imperv_f
      4. Cell assignment = CELL_CENTER
      5. Priority Field = EM_IMPERV
      6. Cellsize = 10 (m)
   2. Multiplied floating grid by 10 million using ArcCatalog -> ArcToolbox -> Spatial Analyst Tools -> Math -> Times
      1. Input raster = p_imperv_f
      2. Constant value 2 = 10,000,000
      3. Output raster = em_imperv_fXe7
   3. Converted floating point grid to integer grid to reduce file size using ArcCatalog -> ArcToolbox -> Spatial Analyst Tools -> Math -> Int
      1. Input raster = em_imperv_fXe7
      2. Output raster = emimperv_mg60
   4. Deleted intermediate floating point grids em_imperv_f and em_imperv_fXe7
2. PSNERP future scenarios are in a UTM Zone 10 (NAD83, meters) coordinate system, so reprojected WADOE polygons (coho basins only) to same coordinate system as PSNERP using
   1. Input Dataset = WADOE_Puget_Sound_au_Albers.shp
   2. Input Coord System = Albers (default from input shapefile)
   3. Output Dataset = wadoe_basins_utm.shp
   4. Output Coord System = NAD_1983_UTM_Zone_10N
3. Converted WADOE polygons to 10m grid using ArcCatalog -> ArcToolbox -> Conversion Tools -> To Raster -> Polygon to Raster
   1. Input Features = wadoe_basins_utm.shp
   2. Value Field = WADOE_ID
   3. Output Raster Dataset = wadoe_utm
   4. Cell assignment = CELL_CENTER
   5. Priority Field = WADOE_ID
   6. Cellsize = 10 (m)
4. Combined three future scenarios with the WADOE subbasins grid using ArcCatalog -> ArcToolbox -> Spatial Analyst Tools -> Local -> Combine
   1. Input Rasters =
      1. Wadoe_utm
      2. Eimperv_mg60
      3. Eimperv_sq60
      4. Eimperv_ug60
   2. Output Raster = eimperv_comb
5. Exported value attribute table (VAT) from eimperv_comb and loaded into Excel
6. Calculated an area weighted mean (AWM) percent imperviousness for each WADOE subbasin for each of the three future scenarios using the following equation:


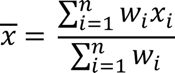


where *x* is the imperviousness value for a given group of grid-cells, and *w* is the number of grid cells that fall within a given site subbasin. Given the fine spatial grain (10 m grid cell size), we did not correct for grid cells that fell on sub-basin boundaries.

Using the output from the spatial analyses we created maps of sub-basins that should be prioritized for conservation based on Δ*Z* values, current percent imperviousness reported by Feist et al. (2017) and predicted future imperviousness from Bolte & Vache (2011). Subbasins that had current imperviousness <10% and Δ*Z* > 0, but were projected to have an imperviousness ≥10% in any of the three future scenarios were tagged as a preservation priority. Subbasins not meeting those criteria were deemed not a preservation priority

**References**

Bolte, J & KB Vache (2011) Envisioning Puget Sound Alternative Futures. Oregon State University, University, OS, Corvallis, OR, 50 p. URL<http://envision.bioe.orst.edu/StudyAreas/PugetSound/PSNERP_Final_Report.pdf>

Bolte, J, KB Vache, C Schwartz, DW Hulse, S Gregory, A Branscomb & C Smith (2010) Oregon State University, Corvallis, OR USA. URL<http://envision.bioe.orst.edu/StudyAreas/PugetSound/> Accessed: 2 April 2019.

WADOE (2011) "Analysis units (AU)" Washington State Department of Ecology, Olympia, WA. URL<https://fortress.wa.gov/ecy/coastalatlas/wc/landingpage.html> Accessed: 7 June 2015.

**Supplemental Tables**

**Table S1. Highest priority sub-basins in need of preservation (**Δ*Z* **>0) vary by conservation metric.** We show the 25 highest priority subbasins, by metric, for the three metrics we quantified (with *M*_crit_ = 0.3 and *𝛂* = 0.95).

| **Priority Rank** | **Coho Sub-Basin ID** | **Coho Habitat (m)** | **Chinook Sub-Basin ID** | **Chinook Habitat (m)** |
| --- | --- | --- | --- | --- |
| 1 | 296 | 36592 | 296 | 36592 |
| 2 | 15 | 26775 | 15 | 22465 |
| 3 | 2095 | 21132 | 319 | 20397 |
| 4 | 319 | 21089 | 63 | 13801 |
| 5 | 2502 | 20973 | 1189 | 13283 |
| 6 | 588 | 17999 | 2095 | 13045 |
| 7 | 2557 | 17249 | 1195 | 12606 |
| 8 | 2293 | 16697 | 329 | 12044 |
| 9 | 1507 | 16721 | 273 | 12099 |
| 10 | 1536 | 16521 | 332 | 11656 |
| 11 | 1953 | 16311 | 1586 | 11616 |
| 12 | 1936 | 15963 | 588 | 11409 |
| 13 | 2306 | 15742 | 2246 | 10325 |
| 14 | 273 | 15500 | 25 | 10367 |
| 15 | 1564 | 15381 | 2477 | 10037 |
| 16 | 2256 | 15200 | 330 | 9921 |
| 17 | 2246 | 15119 | 2025 | 9905 |
| 18 | 63 | 14918 | 1239 | 9830 |
| 19 | 2295 | 14723 | 1192 | 9660 |
| 20 | 1934 | 14408 | 2295 | 9308 |
| 21 | 1586 | 14272 | 271 | 9374 |
| 22 | 25 | 13916 | 1460 | 9366 |
| 23 | 329 | 13709 | 306 | 9313 |
| 24 | 2250 | 14027 | 44 | 8908 |
| 25 | 2025 | 13283 | 2594 | 9063 |

**Table S2.Highest priority sub-basins in need of restoration (**Δ*Z* **<0) vary by conservation metric.** We show the 25 highest priority subbasins, by metric, for the three metrics we quantified (with *M*_crit_ = 0.3 and *𝛂* = 0.95).

| **Priority Rank** | **Coho Sub-Basin ID** | **Coho Habitat (m)** | **Chinook Sub-Basin ID** | **Chinook Habitat (m)** |
| --- | --- | --- | --- | --- |
| 1 | 2089 | 30007 | 2702 | 32553 |
| 2 | 2702 | 32553 | 2087 | 22747 |
| 3 | 2162 | 28445 | 491 | 24516 |
| 4 | 2624 | 25682 | 2624 | 20913 |
| 5 | 2165 | 25222 | 2160 | 19522 |
| 6 | 2158 | 24352 | 462 | 18534 |
| 7 | 2087 | 24222 | 2088 | 17923 |
| 8 | 2161 | 23921 | 497 | 17482 |
| 9 | 937 | 23335 | 328 | 16524 |
| 10 | 2160 | 23094 | 96 | 15273 |
| 11 | 2088 | 22757 | 95 | 14485 |
| 12 | 2766 | 21667 | 26 | 14065 |
| 13 | 2629 | 21303 | 2911 | 13810 |
| 14 | 491 | 21609 | 398 | 14024 |
| 15 | 2902 | 19338 | 937 | 13802 |
| 16 | 497 | 19031 | 2089 | 13197 |
| 17 | 2113 | 19129 | 2094 | 13762 |
| 18 | 2302 | 18581 | 399 | 13582 |
| 19 | 462 | 18534 | 374 | 14221 |
| 20 | 2917 | 19781 | 2327 | 12617 |
| 21 | 2328 | 17637 | 74 | 12487 |
| 22 | 77 | 17490 | 86 | 12809 |
| 23 | 68 | 17416 | 1242 | 12412 |
| 24 | 328 | 17102 | 2082 | 12632 |
| 25 | 26 | 16908 | 2766 | 12543 |

**Supplemental Figures**


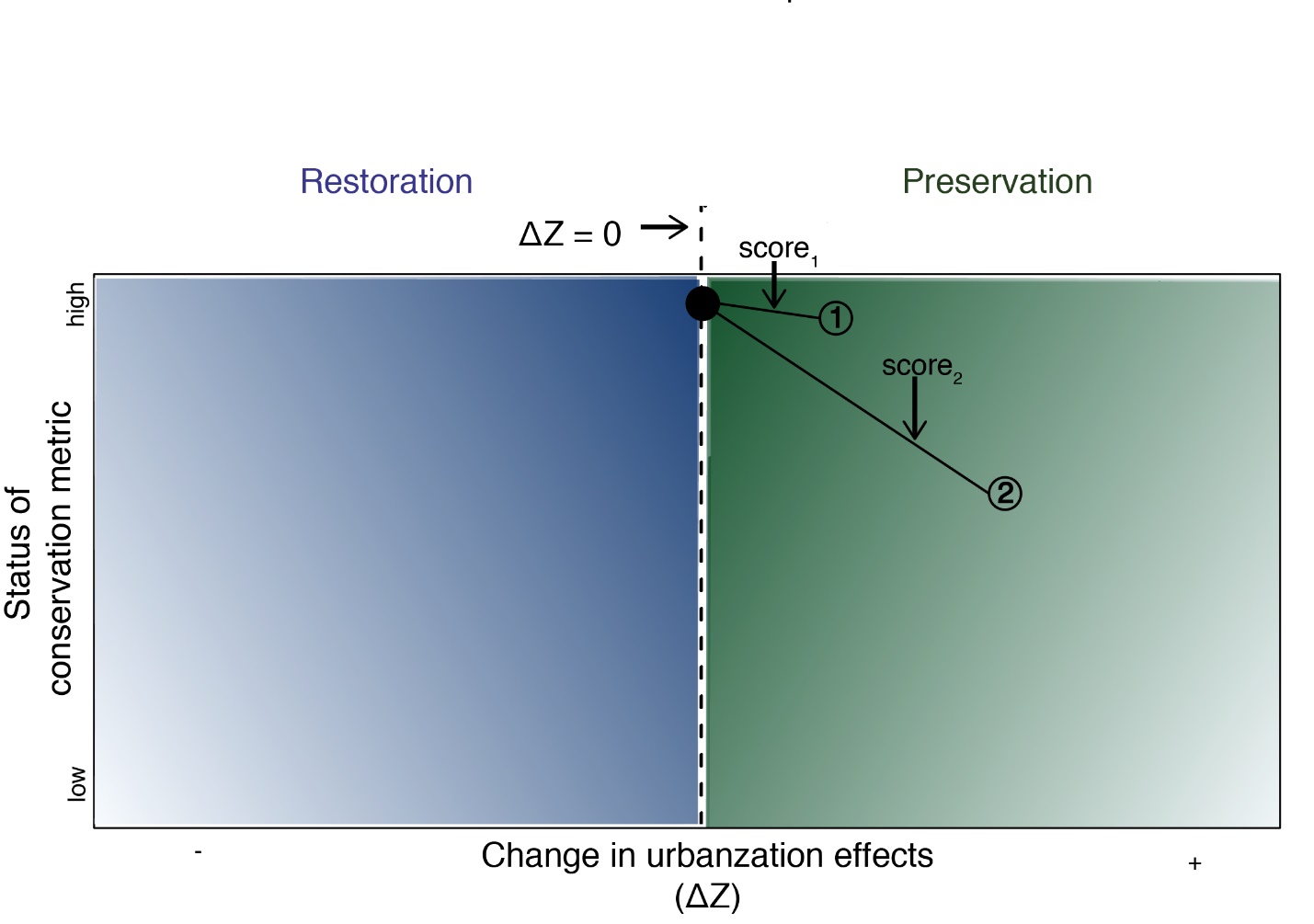


**Figure S1. Prioritization scores** were calculated as the Euclidean distance between conditions at each subbasin (e.g., 1 and 2) and the maximum value of the focal conservation metric at ΔZ = 0 (black circle). For example, subbasin 1 is higher priority (i.e., it has a lower score) than subbasin 2 in the figure above. In other words, ${score}_{1}< {score}_{2}$ because $\mathrm{dist}\left( \left( {\Delta z}_{1}, {metric}_{1} \right), \left( 0, {metric}_{max} \right) \right)<\mathrm{dist}\left( \left( {\Delta z}_{2}, {metric}_{2} \right), \left( 0, {metric}_{max} \right) \right)$. See Appendix 1 for R code.
